# Live Single-Cell Metabolomics With Matrix-Free Laser/Desorption Ionization Mass Spectrometry to Address Microalgal Physiology

**DOI:** 10.3389/fpls.2019.00172

**Published:** 2019-02-18

**Authors:** Tim U. H. Baumeister, Marine Vallet, Filip Kaftan, Aleš Svatoš, Georg Pohnert

**Affiliations:** ^1^Max Planck Fellow Group on Plankton Community Interaction, Max Planck Institute for Chemical Ecology, Jena, Germany; ^2^Research Group Mass Spectrometry/Proteomics, Max Planck Institute for Chemical Ecology, Jena, Germany; ^3^Institute for Inorganic and Analytical Chemistry, Bioorganic Analytics, Friedrich Schiller University Jena, Jena, Germany

**Keywords:** microalgae, single-cell metabolomics, live single cell mass spectrometry, matrix-free laser desorption/ionization-mass spectrometry, multivariate statistics, diatoms *Coscinodiscus* (Bacillariophyceae), chlorophyceae *Haematococcus pluvialis*

## Abstract

Unicellular phototrophic algae can form massive blooms with up to millions of individual cells per milliliter in freshwater and marine ecosystems. Despite the temporal dominance of bloom formers many algal species can co-exist and compete for nutrients and space, creating a complex and diverse community. While microscopy and single cell genomics can address the taxonomic inventory, the cellular metabolome has yet to be thoroughly explored to determine the physiological status of microalgae. This might, however, provide a key to understand the observed species diversity in the homogeneous environment. Here, we introduce an effective, rapid and versatile method to analyze living single cells from aqueous substrata with laser-desorption/ionization mass spectrometry (LDI-MS) using a simple and inexpensive matrix-free support. The cells deposited on a cultivation-medium wetted support are analyzed with minimal disturbance as they remain in their natural viable state until their disruption during LDI-MS. Metabolites desorbed from single cells are analyzed on High-Resolution Mass Spectrometry (HR-MS) using the Orbitrap FT-MS technology to fingerprint cellular chemistry. This live single-cell mass spectrometry (LSC-MS) allows assessing the physiological status and strain-specifics of different microalgae, including marine diatoms and freshwater chlorophytes, at the single-cell level. We further report a reliable and robust data treatment pipeline to perform multivariate statistics on the replicated LSC-MS data. Comparing single cell MS spectra from natural phytoplankton samples and from laboratory strains allows the identification and discrimination of inter and intra-specific metabolic variability and thereby has promising applications in addressing highly complex phytoplankton communities. Notably, the herein described matrix-free live-single-cell LDI-HR-MS approach enables monitoring dynamics of the plankton and might explain why key-players survive, thrive, avoid selective feeding or pathogenic virus and bacteria, while others are overcome and die.

## Introduction

Cell-to-cell heterogeneity is defined by differential metabolic expression resulting in diverse phenotypes in seemingly homogeneous populations (Malviya et al., 2016). The cell performance can vary tremendously within a population in response to environmental cues and other external stimuli. Moreover, cellular responses to intra- or inter-specific infochemicals released by the conspecifics or competing species can be diverse according to the physiological state of the respective cells (Zenobi, 2013). Numerous challenges are faced when addressing the concentration, structural identity and functional role of cellular metabolites (Kuhlisch and Pohnert, 2015). In particular, rapid and high throughput methods are required to cover the dynamics of metabolite expression that is causing substantial fluctuations in concentrations over the course of the development of a culture or bloom (Barofsky et al., 2010; Vidoudez and Pohnert, 2012). Being able to identify and quantify metabolites in single cells, however, will support the prediction of dynamics in large populations of microalgae by assigning fitness status to respective cell cohorts (Acevedo-Trejos et al., 2018).

Complementary analytical tools are paramount to record the cellular heterogeneity but the inventory is rather limited. Several single cell approaches including fluorescence microscopy require labeling experiments which can interfere with the cell physiology (Ettinger and Wittmann, 2014). Other non-destructive methods such as Raman microscopy enable molecular fingerprinting of single cells, but Raman signals are only observed from a few functional groups (Smith et al., 2016). Single-cell mass spectrometry (SC-MS) is one of the foremost strategies to record metabolic profiles of single cells and the cellular metabolic activities. Several SC-MS approaches have been developed, many of which involve significant sample preparation, including, but not limited to, treatment with an organic matrix or evacuation under vacuum. These treatments can lead to cellular degradation or other stress that can trigger wound-activated chemical transformations and alter the metabolic signature of a cell within seconds (Pohnert, 2000). Single cell analysis by ionization under ambient pressure offers the lowest perturbation, granting the analysis within minutes (Comi et al., 2017; Yang et al., 2017; Sun et al., 2018; Zhang and Vertes, 2018).

The sample preparation plays a crucial role in depicting a realistic portrait of the cellular analytes; the methods at disposal include suction of the cell content by micro capillary or extraction of the metabolites by nanomanipulation (Fujii et al., 2015; Lee et al., 2016). However, these approaches are technically challenging and often lack sensitivity. Laser desorption/ionization (LDI) strategies provide an alternative, where analytes from single cells can be ionized using a pulsed UV-laser. Cells are thereby destroyed and metabolites are simultaneously ionized. In matrix-assisted laser desorption/ionization mass spectrometry (MALDI-MS) approaches, this process is supported by an externally added matrix. However, matrix signals dominate the low molecular range of the mass spectra. Two matrix-free platforms have been optimized for LDI-MS applications. Nano post arrays (NAPA) provide nanostructured surfaces that support ionization as demonstrated, e.g., in the investigation of yeast cell metabolites (Walker et al., 2012). If cells themselves are patterned, this property can be used for a direct imaging using a pulsed laser without the support of matrix or additional structured surfaces (Jaschinski et al., 2014). Especially cells from phytoplankton, like diatoms with nanopatterned silicate cell walls fulfill this pre-requisite. Furthermore, phytoplankton cells inherit significant amounts of chromophores, like chlorophyll *a* (Chl *a*) and carotenes that can be easily ionized through photoionization by a UV laser (Suzuki et al., 2009; Jaschinski et al., 2014; Mandal et al., 2019). The aforementioned techniques require evacuation of the sample to vacuum before profiling of the cells. With the present contribution, we overcome this limitation and introduce an ambient pressure matrix-free ionization of cells in their native state. We proceed to analyze single cells of the freshwater algal model *Haematococcus pluvialis* and of the marine diatom *Coscinodiscus granii* with LDI-HR-MS, by decrypting changes in their cellular metabolome during aging and nutrient-depletion. The present method discriminated natural and laboratory strains of diatoms *Coscinodiscus granii*, hence opening perspectives in taxonomic identification at the single cell level during natural phytoplankton blooms.

## Materials and Methods

### Materials

For diatom cell wall preparation fresh diatom cultures (30 mL, see below) were centrifuged for 15 min at 3000 relative centrifugal force and clean empty bio mineralized diatom cell walls (frustules) were obtained by treating the pellet in 1 mL of hydrogen peroxide (1.5%) at 90°C for 2 h, followed by three washing steps of the cell pellet with acetonitrile/water (1:1) (Aitken et al., 2016). The empty shells were stored in pure ethanol at 4°C. Analytical standards Chl *a*, fucoxanthin, β-carotene, astaxanthin (Atx) and chemicals, if not mentioned otherwise were obtained from Merck (Darmstadt, Germany). Dimethylsulfoniopropionate (DMSP) was synthesized according to a published procedure (Chambers et al., 1987). Standards were prepared in UPLC grade pure ethanol or chloroform at 1 mg × mL^-1^ and 2 μL were spotted on cleaned diatom cell walls (frustules) or on humid GF/C filter stacks, air-dried and analyzed as described above.

### Cultivation of Algae

Heterogeneous phytoplankton samples were collected in German coastal waters during a bloom in August 2016. *Coscinodiscus granii* (isolate Helg2016) was identified and selected based on key taxonomic criteria (Lundholm et al., 2002; Zimmermann et al., 2011; Kesseler, 2015; Wang et al., 2015). Cultures were grown in half strength Guillard’s (F/2) enrichment medium prepared with natural sea water (ATI, Hamm, Germany). Monoclonal strains from culture collections *Coscinodiscus granii* strain SCCAP-K1834 (isolation in Denmark in 2012) and *Haematococcus pluvialis* strain SAG 192.80 were maintained in artificial seawater medium and freshwater Blue-Green (BG11) medium (Stanier et al., 1971), respectively. To study the influence of aging and nutrient depletion on the cellular metabolome, the algae under study were cultivated for 15 days under daylight fluorescent lamps (irradiance 100 mE × m^-2^ × s^-1^) with a 14 h photoperiod coupled to a thermo-regulated cycle (16°C/12°C day/night). The growth was monitored by counting with a Sedgewick-Rafter chamber (Pyser Optics, Kent, United Kingdom) every second day. For *Haematococcus*, cultures were left incubating for 15 days until observing cells in “red” phenotype. Pictures were taken with an inverted microscope Axiovert2plus coupled to AxioCam MRc 5 camera (Carl Zeiss AG, Oberkochen, Germany). One culture per species or strain was used to randomly recover a minimum of 20 cells for single cell profiling.

### Profiling of Living Single Cells by LDI-HR-MS

Prior to single cell analysis GF/C glass fiber filters (Whatman, Maidstone, United Kingdom) were cut in rectangles of 15 × 12 mm and washed thrice with methanol (LiChrosolv hypergrade for LC-MS, Merck, Germany) and *n*-hexane (Rotipuran ≥99% p.a., Roth, Germany). Algae cells were manually collected from cultures with a Pasteur pipette and promptly applied to a three layered stack of washed GF/C filters wetted beforehand by adding 50 μL of F2 medium homogeneously over the filter. Filters with cells were placed on a clean microscopic glass slide (15 × 12 mm) that was fixed on a holder. All samples were analyzed via an AP-SMALDI (AP-SMALDI10, TransMIT, Gießen, Germany) ion source equipped with an UV (337 nm) nitrogen laser (LTB MNL-106, LTB, Germany) with a spot size of 10 μm. The AP-SMALDI ion source was coupled to the mass spectrometer Q-Exactive Plus (Thermo Fisher Scientific, Bremen, Germany), which provided high resolution mass spectra. Data were collected using an Xcalibur software version 2.8 Build 1824 (Thermo Fisher Scientific, Bremen, Germany). Samples were analyzed in both polarities with the number of laser shots per spot set to 30 (approximately 1.5 μJ × shot^-1^) within the laser frequency of 60 Hz. Mass spectra were recorded in the mass range from *m/z* 100–1000 Da with the peak resolution of 70,000. The analysis of one single cell yielded a live single-cell mass spectrum (LSC-MS) and 20 individual cells were analyzed in each sample. Raw data was converted into the netCDF format using the Thermo File Converter. Spectra of media blanks were obtained before each experiment. Spectra from different cells (*N* > 20) per treatment (species, strain, age) were collected from single cells that were selected visually. Datasets and the script employed are available upon request.

### Significant Features Analysis

Converted raw data was pre-processed in R (R Core Team, 2018), using the packages *MALDIquant*, and *MALDIquantForeign* (Gibb and Strimmer, 2012). Noise was estimated via median intensity and signals below a signal-to-noise ratio of 5 were removed from further processing. The peaks were aligned and those detected in the blank medium were excluded from the peak matrix. Peak intensities were TIC normalized and Pareto scaled. Only signals occurring in more than half of the samples of the group (nutrients status, age or strain, respectively) were selected and processed. The MS spectra were recorded in positive polarity. For the quantile-quantile plots, TIC normalized and Pareto scaled peak intensities of Chl *a* (*m*/*z* 892.5345), β-carotene (*m*/*z* 536.4371), and fucoxanthin (*m*/*z* 658.4214) of the Helg2016 and SCCAP-K1834 (Day 1) data were selected. Quantile-quantile plots were created using the R *stats* package to visualize if the data were normally distributed. Normality test was assessed with the Shapiro-Wilk test (R Core Team, 2018). Unsupervised principal component analysis (PCA) and partial least squares discriminant analysis (PLS-DA) were performed to highlight metabolic variations between LSC-MS profiles, using the *MetaboanalystR* package (Gromski et al., 2015; Xia et al., 2015). After PLS-DA calculation, permutation test and cross validation (CV) were performed to verify the significance of the test and validate the selected model, respectively. Significant features were determined by calculating the sum of squares of the PLS loadings for each component, giving a variable of importance in projection (VIP score), including 10 top features without taking into account an absolute threshold. To assess the significance of class discrimination, a permutation test on 1000 simulations based on prediction accuracy was performed. Further validation was inferred with the Random Forest algorithm to construct classification trees which were grown by random sample selection from a bootstrap sample at each branch. The random forest provided an OOB (out-of-bag) error to obtain an unbiased estimate of the classification error.

## Results and Discussion

### Mass Spectrometry Profiling of Living Single Cells With LDI-MS

**FIGURE 1:**
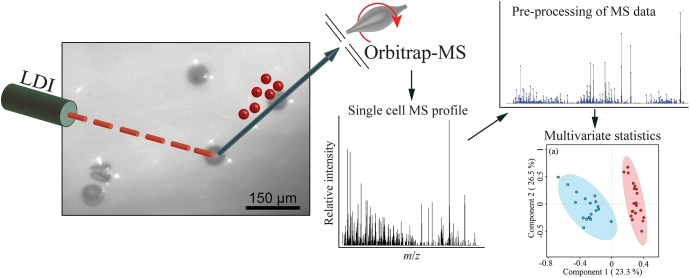
Scheme for acquisition and data treatment of single cell MS profiling of unicellular algae.

**FIGURE 2:**
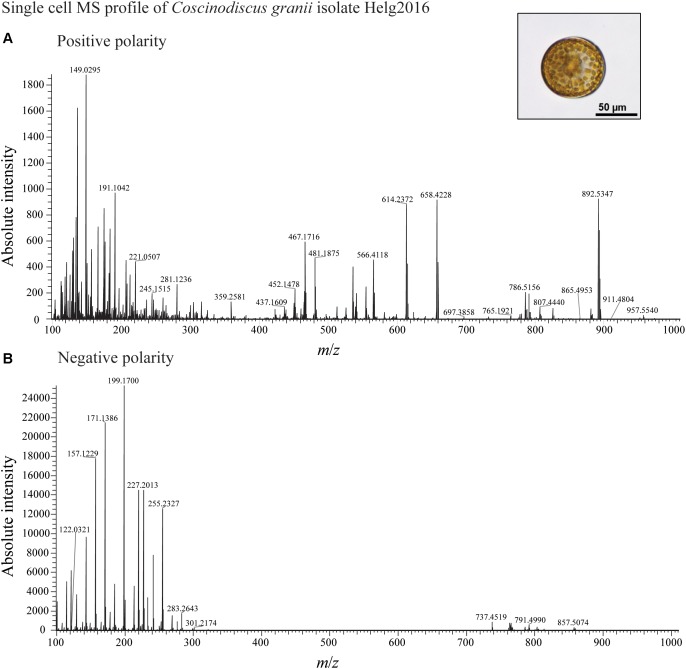
LDI-MS profiling of a single cell of diatom *C. granii* acquired in positive **(A)** and **(B)** negative mode.

**FIGURE 3:**
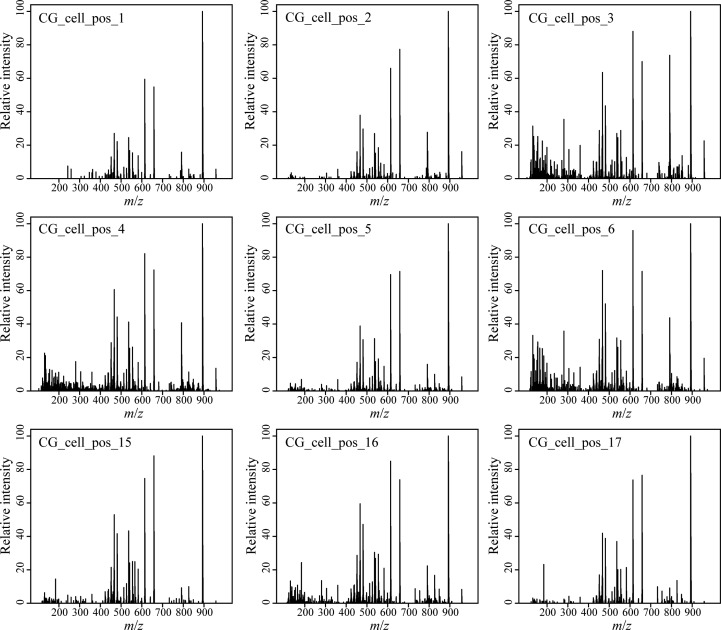
Blank subtracted LSC-MS spectra of nine different consecutively analyzed cells of the diatom *Coscinodiscus granii* isolate Helg2016 recovered randomly from one culture at the same incubation time.

**FIGURE 4:**
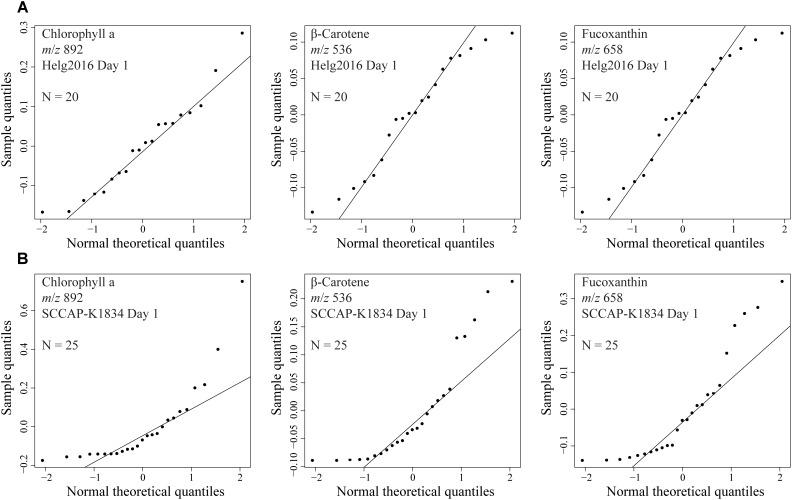
Quantile-Quantile plots to assess if observed peak intensities from signals obtained from several analyzed microalgal cells follow a normal distribution. Selected signals were chlorophyll a (*m*/*z* 892), fucoxanthin (*m*/*z* 658), and β-carotene (*m*/*z* 536). Diatom cells from the same culture at day 1 early growth of *Coscinodiscus granii* **(A)** isolate Helg2016 (*N* = 20) and **(B)** strain SCCAP-K1834 (*N* = 25).

We developed a rapid and effective method to obtain the chemical profile of living single cells without prior preparation or application of matrix. As described in Figure 1, unicellular microalgae were sampled from their culture by pipetting cells with their culture medium on a GF/C filter. These were then directly analyzed with ambient pressure LDI-HR-MS in both polarities. Single cells in size above 10 μm were easily visualized on GF/C filter by a mounted AP-SMALDI camera. This enables visual targeting the laser directly at a single cell. We did not evaluate the minimum cell size, for the detection of MS-spectra which will be below this value. The laser irradiation completely disrupted the cell within a minute and desorbed/ionized molecules formed cell-specific chemical patterns (Figure 2, 3). After that process, no visually recognizable cellular material was left on the GF/C filter. The analysis in positive polarity yielded higher number of mass peaks than in negative mode (Figure 2). Raw spectra were processed with an in-house script based on the *MALDIquant* R package, reducing the background noise signal, removing the signals from the culture medium, and including a total ion current (TIC) normalization as recommended when working with MALDI-MS (Gibb and Strimmer, 2012; Emara et al., 2017). Since application of internal standard is not feasible as it would induce stress in the living cell, the relative quantification based on TIC normalization was selected. To test the reproducibility of our MS profiling, the chemical signatures of single algal cells recovered from one culture of *C. granii* isolate Helg2016 and one culture of a different strain of the same species (SCCAP-K1834) were acquired (Figure 3 shows exemplary processed LSC-MS spectra of nine cells). A total of 326 and 400 peaks were recovered, respectively, from the LSC-MS spectra. Data from *C. granii* isolate Helg2016 and strain SCCAP-K1834 were compared by a PCA and did not display any obvious pattern (Supplementary Figure S1). Furthermore, three prominent signals (Chl *a*, fucoxanthin, and β-carotene) were chosen from the dataset and tested if the respective intensities followed a normal distribution using quantile-quantile plots and the Shapiro-Wilk test (Figure 4 and Supplementary Table S1). A deviation from the normal distribution would indicate heterogeneity in the tested cell population. The signal intensities of the chosen photosynthetic pigments obtained from isolate Helg2016 were normally distributed, but not from strain SCCAP-K1834. Cells in cultures of the strain SCCAP-K1834 were very heterogeneous in size (Supplementary Figure S2), which may explain the observed variability of photosynthetic pigments. Indeed, the size of the cells substantially influences the pigment content in *Coscinodiscus granii* (Yan et al., 2018). This result indicates that our LSC-MS approach may be also able to address the single cell heterogeneity within a population.

HR-MS allows assignment of certain heteroatoms, e.g., N, O, S, and halogens due to characteristic mass defects and the characteristic relative intensity of isotope peaks. Only at high molecular weight isotopic deconvolution is not straightforward, as illustrated in the [M+H]^+^ of Chl *a* (*m*/*z* 893.5371) which comprises the isotopolog containing ^25^Mg ^13^C, ^15^N. To simplify readability of this text, nominal mass-charge ratios will be used from here on (compare tables for accurate mass-charge ratios). MS^2^ analysis could not be achieved from signals from single cells due to insufficient ion intensities. However, the MS^2^ fragmentation of commercially available analytes that were pipetted on empty frustules of *C. granii* could be achieved (Jaschinski et al., 2014) to confirm the identity of cellular and commercial metabolites. Furthermore, analysis of extracts in LC-MS and LC-MS-MS can help in the identification of detected metabolites. With this approach, main constituents of the algal cells within a broad mass range were identified.

The LSC-MS signals in positive polarity for photosynthetic pigments, including Chl *a*, fucoxanthin and β-carotene, dominate the chemical profile of the unicellular algal cells. Interestingly, fragments of Chl *a* were observed (*m*/*z* 614, 452, 467, 481), suggesting in-source fragmentation. This in-source fragmentation could not be avoided as no signal could be detected anymore when reducing the laser energy. In particular photosynthetic algal pigments that are commonly used for algal taxonomic characterization could be identified (Table 1). It is hence assumed that signals in the *m*/*z* 200–500 range may arise from fragments or different adducts. Common techniques employ liquid- or gas-chromatography and Fourier-transform infrared spectroscopy profiling of pigments (Biller and Ross, 2014; Meng et al., 2014; Agirbas et al., 2015), but our LSC-MS approach offers direct analysis and higher resolution of the pigment composition at the single cell level. The multifunctional zwitterionic metabolite DMSP (*m*/*z* 135) was also detected and its identity was further verified in cells extract of *C. granii* with LC-MS as described in Spielmeyer and Pohnert (2010) (Supplementary Figure S3).

**Table 1:** Summary of measured compounds and detected signals with LDI-MS.

Analytes	Monoisotopic ion molecular mass (u)	Measured ion (*m*/*z*) MS1 (positive polarity)	Measured ion (*m*/*z*) MS2 (positive polarity)	
			Precursor	Fragment
Chlorophyll a	892.5342	892.5352	892.5357	614.2366, 481.1866
Fucoxanthin	658.4227	658.4226	658.4201	515.3516, 473.3516
β-carotene	536.4377	536.4370		
Astaxanthin	596.3860	596.3856	597.1625	582.1386
DMSP	135.0474	135.0475		


### Strain- and Age-Specific Profiling of Diatoms *C. granii* Unraveled by Matrix-Free LDI-MS

**FIGURE 5:**
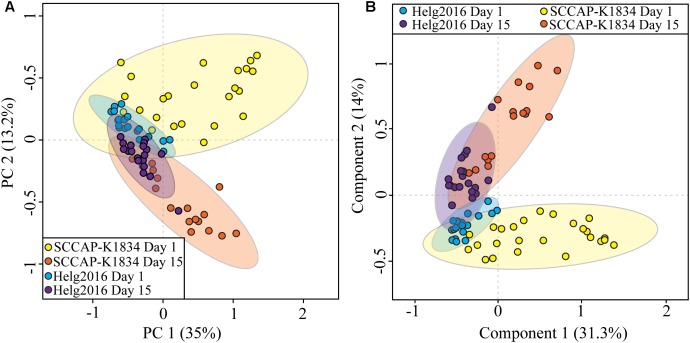
Statistical comparison of LSC-MS profiles of *Coscinodiscus granii* strain SCCAP-K1834 and isolate Helg2016 recovered from early (Day 1) and late (Day 15) growth stages. **(A)** PCA score plot **(B)** PLS-DA score plots displaying dissimilarities between LSC-MS profiles of young (day 1 early exponential growth phase) and old (day 15 early stationary growth phase) cells of *C. granii*. The LSC-MS profiles of both strains are included in the analysis. The ellipses represented the 95% confidence region estimated by the statistical calculation.

Monoclonal *C. granii* (strain SCCAP-K1834) and the non-clonal isolate (Helg2016) sampled during a bloom in August 2016 in German coastal waters were grown in triplicate in their respective medium over 15 days. *C. granii* isolate (Helg2016) reached a cell count of 200 cells × mL^-1^ in six days while three times higher cell density was obtained for the strain SCCAP-K1834 (Supplementary Figure S2). Single cells were isolated and profiled with LSC-MS to determine metabolic differences between strains and to compare cells in logarithmic (day 1) and stationary (day 15) growth phases. The peak matrix contained 595 peaks which were found at least in 50% of the samples after background subtraction. An unbiased PCA was performed on processed LSC-MS profiles and explained 48.2% of total variance (Figure 5A). The strain clearly explained most of the variance observed (PC1 35%) whereas the cell age (PC2) accounted for 13.2% of variance. To further assess our ability to distinguish cell age with the LSC-MS method, a PLS-DA was carried out on the LSC-MS profiles of young and old *C. granii* cells sampled at early exponential and early stationary growth phases (Figure 5B). The cumulative variance was 45.3%, while most of the variance was explained by component 1 (31.5%). The model fitness was confirmed by a 1000 permutation test (*p*-value <0.001) and the predictive ability to assign a “young” or “old” age status was assessed and visualized by cross-validation test (Supplementary Figure S4). An *in silico* classification test was performed (random forest) to confirm the reliability of assigning one data set from a single cell to the right class with out-of-bag errors (data not shown). These tests demonstrated that there was a very low percentage of overall error of 4.5% in assigning a group (old, young, strain) to a cell by its sole LSC-MS profile.

To identify metabolites related to cell aging or strain-specific markers, the variables of importance in projection (VIP score) from the PLS-DA analysis were calculated, leading to features that were tentatively assigned by matching HR-MS spectra. Several metabolites could be confirmed with standards or attributed to entries in the database. Among them, pigments fucoxanthin (*m*/*z* 658) and Chl *a* (*m*/*z* 892) were absent from LSC-MS profiling of old *Coscinodiscus granii* in stationary phase, but carotene (*m*/*z* 536) and alloxanthin (*m*/*z* 566) were still detected. Pigment reduction has been previously observed in diatoms under nitrogen deficiency (Thomas and Dodson, 1972; Alipanah et al., 2015) consistent with the phenotype observed here in *C. granii* when reaching the stationary phase. It is also known that some diatoms can increase their production of defensive polyunsaturated aldehydes in aged and nutrient-depleted cultures (Alipanah et al., 2015; Sayanova et al., 2017). These metabolites are produced from lipids that are initially lysed to release free fatty acids for further processing. In agreement, some metabolites were putatively assigned to a neutral glycosphingolipid (*m*/*z* 732.5613) and glycerophosphoserines (*m*/*z* 826.5358, 791.4664).

### LSC-MS Profiling of *H. pluvialis* Response to Nutrient Depletion

**FIGURE 6:**
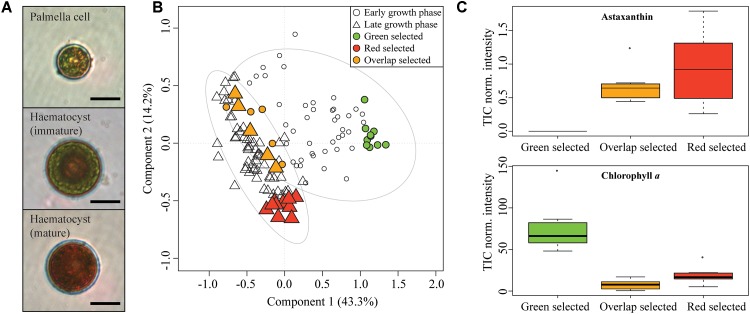
**(A)** Microscopy observations of a palmella cell (green stage), immature and mature haematocyst (red stage). Scale bar 10 μm. **(B)** PLS-DA score plot of LSC-MS profiles of *H. pluvialis* cells in early and late growth stage. The ellipses represented the 95% confidence region estimated by the statistical calculation. **(C)** Comparison of pigments content (represented as TIC normalized intensity) in selected group of LSC-MS spectra of red, green and overlap cells.

The commercially important chlorophyceae *Haematoccocus pluvialis* strain SAG 192.80 was selected as model to study the effect of nutrient stress on the metabolome at the single-cell level. The alga evolves from a motile, small, green cell to a large, deep red colored cell upon nutrient depletion (Shah et al., 2016). The palmella cells (A) belong to the green vegetative phase. Later red hematocysts are formed (Figure 6A). Cultures of *H. pluvialis* were grown until nutrient exhaustion for 15 days and random cells in early and late cultures (*N* > 20) were profiled with LSC-MS. Partial Least Square Discriminant Analysis (PLS-DA) was carried out to compare the LSC-MS profiles of early and late stage cultures (Figure 6B). A total explained variance of 57.4% was observed and the features in the component 1 (phenotype early and late) explained 43.3% of the variance. Algal populations of *H. pluvialis* are usually asynchronous with cells at various encystment stages, and hence several phenotypes can be simultaneously observed in one culture. This contributes to the non-complete separation of the groups in the PLS-DA (Figure 6B). The model fitness was assessed by the 1000 permutation test (*p*-value <0.001). Significant features were associated with either phenotype “early” or “late.” The early palmella cells are characterized by the presence of Chl *a* (*m*/*z* 892) and other masses (*m*/*z* 184, 305, 335, 526, 555, 568, 481, 559, 690, 423) for which the associated metabolites were not further identified. The late cultures are dominated by hematocyst cells and were distinguished by their xanthophyll content (*m*/*z* 550, 573) and two metabolites tentatively assigned to the class of lipids (*m*/*z* 603, 459). *H. pluvialis* is known to produce the antioxidant carotenoid astaxanthin through conversion of carotene in multiple steps, generating intermediates such as pheonicaxanthin, cryptoxanthin or adonixanthin (Lemoine and Schoefs, 2010; Shah et al., 2016). In accordance with the observed pigmentation astaxanthin (*m*/*z* 596) is up-regulated in the “red” stage (Figure 6C). The content Astaxanthin and Chl *a* in red and green cells were found significantly different by a Kruskal-Wallis one-way ANOVA (Atx: *H* = 20.545, Chl *a*: *H* = 22.516, *p*-value <0.001). It is remarkable that only the analysis of 20 cells is sufficient for a statistically sound physiological characterization. The loadings plot for the PLS-DA reveals Chl *a* (*m*/*z* 892 and related fragments *m*/*z* 467, 614, 481) as discriminating the early phase (Supplementary Figure S5). This was confirmed by paired *t*-test (Supplementary Figure S6). The ability to identify high astaxanthin producing single *H. pluvialis* cells might in the future enable an analytics-supported breeding of commercially relevant pigment producers and reduce the necessity for genetic engineering (Steinbrenner and Sandmann, 2006).

Together with an automatized single cell sorting, this presented LSC-MS method is a powerful tool to address the taxonomic diversity and health status of phytoplankton communities in aquatic ecosystems.

## Conclusion

We are now able to profile the metabolome of microalgae (diatoms and chlorophyceae) and to discriminate the physiological status at the single cell level. This cellular profiling of unicellular bloom-forming algae was achieved by single-cell metabolomics using a matrix-free live-single cell LDI-SC-HR-MS approach. Sample preparation ensured that analyzed cells were in native state just until the data collection. The statistical pipeline on MS data permits to discriminate cells in different degree of nutrient-depletion, age and taxonomy. Our method is a straightforward approach to analyze live single cells using ambient ionization mass spectrometry conditions and opens up new avenues of research.

## Author Contributions

FK developed here presented LSC-MS method including sample support and cell handling before the LDI-HR-MS analysis. MV maintained the algae collection and designed the biological experiments. TB developed the data analysis pipeline. MV and TB analyzed the data and wrote the manuscript with the help of GP and AS. All authors conceived this study and approved the manuscript.

## Conflict of Interest Statement

The authors declare that the research was conducted in the absence of any commercial or financial relationships that could be construed as a potential conflict of interest.

## References

[B1] Acevedo-Trejos E. Maranon E. Merico A. (2018). Phytoplankton size diversity and ecosystem function relationships across oceanic regions. *Proc. Biol. Sci.* 285:20180621. 10.1098/rspb.2018.0621 29794050 PMC5998115

[B2] Agirbas E. Feyzioglu A. M. Kopuz U. Llewellyn C. A. (2015). Phytoplankton community composition in the south-east black sea determined with pigments measured by HPLC-CHEMTAX analyses and microscopy cell counts. *J. Mar. Biol. Assoc. U.K.* 95 35–52. 10.1017/S0025315414001040

[B3] Aitken Z. H. Shi L. Reynolds S. T. Thaulow C. Greer J. R. (2016). Microstructure provides insights into evolutionary design and resilience of *Coscinodiscus* sp. *frustule*. *Proc. Natl. Acad. Sci. U.S.A.* 113 2017–2022. 10.1073/pnas.1519790113 26858446 PMC4776537

[B4] Alipanah L. Rohloff J. Winge P. Bones S. M. Brembu T. (2015). Whole-cell response to nitrogen deprivation in the diatom *Phaeodactylum tricornutum*. *J. Exp. Bot.* 66 6281–6296. 10.1093/jxb/erv340 26163699 PMC4588885

[B5] Barofsky A. Simonelli P. Vidoudez C. Troedsson C. Nejstgaard J. C. Jakobsen H. H. (2010). Growth phase of the diatom *Skeletonema marinoi* influences the metabolic profile of the cells and the selective feeding of the copepod *Calanus* spp. *J. Plankton Res.* 32 263–272. 10.1093/plankt/fbp121

[B6] Biller P. Ross A. B. (2014). Pyrolysis GC–MS as a novel analysis technique to determine the biochemical composition of microalgae. *Algal Res.* 6(Part A), 91–97. 10.1016/j.algal.2014.09.009

[B7] Chambers S. T. Kunin C. M. Miller D. Hamada A. (1987). Dimethylthetin can substitute for glycine betaine as an osmoprotectant molecule for Escherichia coli. *J. Bacteriol.* 169 4845–4847. 10.1128/jb.169.10.4845-4847 3308858 PMC213866

[B8] Comi T. J. Do T. D. Rubakhin S. S. Sweedler J. V. (2017). Categorizing cells on the basis of their chemical profiles: progress in single-cell mass spectrometry. *J. Am. Chem. Soc.* 139 3920–3929. 10.1021/jacs.6b12822 28135079 PMC5364434

[B9] R Core Team (2018). *R: A Language and Environment for Statistical Computing*. Vienna: R Foundation for Statistical Computing.

[B10] Emara S. Amer S. Ali A. Abouleila Y. Oga A. Masujima T. (2017). “Single-Cell Metabolomics,” in *Metabolomics: From Fundamentals to Clinical Applications*, ed. Sussulini A. (Cham: Springer International Publishing),323–343.

[B11] Ettinger A. Wittmann T. (2014). Fluorescence live cell imaging. *Methods Cell Biol.* 123 77–94. 10.1016/B978-0-12-420138-5.00005-7 24974023 PMC4198327

[B12] Fujii T. Matsuda S. Tejedor M. L. Esaki T. Sakane I. Mizuno H. (2015). Direct metabolomics for plant cells by live single-cell mass spectrometry. *Nat. Protoc.* 10 1445–1456. 10.1038/nprot.2015.084 26313480

[B13] Gibb S. Strimmer K. (2012). MALDIquant: a versatile R package for the analysis of mass spectrometry data. *Bioinformatics* 28 2270–2271. 10.1093/bioinformatics/bts447 22796955

[B14] Gromski P. S. Muhamadali H. Ellis D. I. Xu Y. Correa E. Turner M. L. (2015). A tutorial review: metabolomics and partial least squares-discriminant analysis – a marriage of convenience or a shotgun wedding. *Anal. Chim. Acta* 879(Suppl. C), 10–23. 10.1016/j.aca.2015.02.012 26002472

[B15] Jaschinski T. Helfrich E. J. N. Bock C. Wolfram S. Svatoš A. Hertweck C. (2014). Matrix-free single-cell LDI-MS investigations of the diatoms *Coscinodiscus granii* and *Thalassiosira pseudonana*. *J. Mass Spectr.* 49 136–144. 10.1002/jms.3316 24677306

[B16] Kesseler H. (2015). The inorganic chemical composition of the cell sap of *Coscinodiscus granii* (Bacillariophyceae, Centrales). *Helgolän. Wiss. Meeresuntersuchung.* 26 481–489. 10.1007/bf01627628

[B17] Kuhlisch C. Pohnert G. (2015). Metabolomics in chemical ecology. *Nat. Prod. Rep.* 32 937–955. 10.1039/c5np00003c 25926134

[B18] Lee J. K. Jansson E. T. Nam H. G. Zare R. N. (2016). High-resolution live-cell imaging and analysis by laser desorption/ionization droplet delivery mass spectrometry. *Anal. Chem.* 88 5453–5461. 10.1021/acs.analchem.6b00881 27110027 PMC5446058

[B19] Lemoine Y. Schoefs B. (2010). Secondary ketocarotenoid astaxanthin biosynthesis in algae: a multifunctional response to stress. *Photosynth. Res.* 106 155–177. 10.1007/s11120-010-9583 20706789

[B20] Lundholm N. Daugbjerg N. Moestrup Ø (2002). Phylogeny of the Bacillariaceae with emphasis on the genus *Pseudo-nitzschia* (Bacillariophyceae) based on partial LSU rDNA. *Eur. J. Phycol.* 37 115–134. 10.1017/S096702620100347X

[B21] Malviya S. Scalco E. Audic S. Vincent F. Veluchamy A. Poulain J. (2016). Insights into global diatom distribution and diversity in the world’s ocean. *Proc. Natl. Acad. Sci. U.S.A.* 113 E1516–E1525. 10.1073/pnas.1509523113 26929361 PMC4801293

[B22] Mandal A. Singha M. Addy P. S. Basak A. (2019). Laser desorption ionization mass spectrometry: Recent progress in matrix-free an label-assisted techniques. *Mass Spectrometry Rev.* 38 3–21. 10.1002/mas.21545 29029360

[B23] Meng Y. Yao C. Xue S. Yang H. (2014). Application of fourier transform infrared (FT-IR) spectroscopy in determination of microalgal compositions. *Bioresour. Technol.* 151 347–354. 10.1016/j.biortech.2013.10.064 24262844

[B24] Pohnert G. (2000). Wound-activated chemical defense in unicellular planktonic algae. *Angew. Chem. Int. Ed.* 39 4352–4354. 10.1002/1521-3773(20001201)39:23<4352::AID-ANIE4352>3.0.CO;2-U 29711885

[B25] Sayanova O. Mimouni V. Ulmann L. Morant-Manceau A. Pasquet V. Schoefs B. (2017). Modulation of lipid biosynthesis by stress in diatoms. *Philos. Trans. R. Soc. Lond. B Biol. Sci.* 372:20160407. 10.1098/rstb.2016.0407 28717017 PMC5516116

[B26] Shah M. R. Liang Y. Cheng J. J. Daroch M. (2016). Astaxanthin-producing green microalga *Haematococcus pluvialis*: from single cell to high value commercial products. *Front. Plant Sci.* 7:531. 10.3389/fpls.2016.00531 27200009 PMC4848535

[B27] Smith R. Wright K. L. Ashton L. (2016). Raman spectroscopy: an evolving technique for live cell studies. *Analyst* 141 3590–3600. 10.1039/C6AN00152A 27072718

[B28] Spielmeyer A. Pohnert G. (2010). Direct quantification of dimethylsulfoniopropionate (DMSP) with hydrophilic interaction liquid chromatography/mass spectrometry. *J. Chromatogr.* 878 3238–3242. 10.1016/j.jchromb.2010.09.031 21030323

[B29] Stanier R. Y. Kunisawa R. Mandel M. Cohen-Bazire G. (1971). Purification and properties of unicellular blue-green algae (order Chroococcales). *Bacteriol. Rev.* 35 171–205. 4998365 10.1128/br.35.2.171-205.1971 PMC378380

[B30] Sun M. Yang Z. Wawrik B. (2018). Metabolomic fingerprints of individual algal cells using the single-probe mass spectrometry technique. *Front. Plant Sci.* 9:571. 10.3389/fpls.2018.00571 29760716 PMC5936784

[B31] Steinbrenner J. Sandmann G. (2006). Transformation of the green alga *Haematococcus pluvialis* with a phytoene desaturase for accelerated astaxanthin biosynthesis. *Appl. Environ. Microbiol.* 72 7477–7484. 10.1128/AEM.01461-06 17012596 PMC1694260

[B32] Suzuki T. Midonoya H. Shioi Y. (2009). Analysis of chlorophylls and their derivatives by matrix-assisted laser desorption/ionization-time-of-flight mass spectrometry. *Anal. Biochem.* 390 57–62. 10.1016/j.ab.2009.04.005 19364490

[B33] Thomas W. H. Dodson A. N. (1972). On nitrogen deficiency in tropical pacific oceanic phytoplankton of a chemostat-grown diatom. *Limnol. Oceanogr.* 17 515–523. 10.4319/lo.1972.17.4.0515

[B34] Vidoudez C. Pohnert G. (2012). Comparative metabolomics of the diatom *Skeletonema marinoi* in different growth phases. *Metabolomics* 8 654–669. 10.1007/s11306-011-0356

[B35] Walker B. N. Stolee J. A. Vertes A. (2012). Nanophotonic ionization for ultratrace and single-cell analysis by mass spectrometry. *Anal. Chem.* 84 7756–7762. 10.1021/ac301238k 22881122

[B36] Wang X.-C. Liu C. Huang L. Bengtsson-Palme J. Chen H. Zhang J. H. (2015). ITS1: a DNA barcode better than ITS2 in eukaryotes? *Mol. Ecol. Resour.* 15 573–586. 10.1111/1755-0998.12325 25187125

[B37] Xia J. Sinelnikov I. V. Han B. Wishart D. S. (2015). MetaboAnalyst 3.0—making metabolomics more meaningful. *Nucleic Acids Res.* 43 W251–W257. 10.1093/nar/gkv380 25897128 PMC4489235

[B38] Yan D. Beardall J. Gao K. (2018). Variation in cell size of the diatom *Coscinodiscus granii* influences photosynthetic performance and growth. *Photosynth. Res.* 137 41–52. 10.1007/s11120-017-0476-6 29322482

[B39] Yang Y. Huang Y. Wu J. Liu N. Deng J. Luan T. (2017). Single-cell analysis by ambient mass spectrometry. *TrAC Trends Anal. Chem.* 90 14–26. 10.1016/j.trac.2017.02.009

[B40] Zenobi R. (2013). Single-cell metabolomics: analytical and biological perspectives. *Science* 342:1243259. 10.1126/science.1243259 24311695

[B41] Zhang L. Vertes A. (2018). Single-cell mass spectrometry approaches to explore cellular heterogeneity. *Angew. Chem. Int. Ed.* 57 4466–4477. 10.1002/anie.201709719 29218763

[B42] Zimmermann J. Jahn R. Gemeinholzer B. (2011). Barcoding diatoms: evaluation of the V4 subregion on the 18S rRNA gene, including new primers and protocols. *Org. Divers. Evol.* 11 173–192. 10.1007/s13127-011-0050-6

