# Supplementary material for: Live Single-Cell Metabolomics With Matrix-Free Laser/Desorption Ionization Mass Spectrometry to Address Microalgal Physiology
Source: Front Plant Sci. 2019 Feb 18;10:172. doi: 10.3389/fpls.2019.00172 (PMC6387974; doi:10.3389/fpls.2019.00172)

Supplementary Material

Single cell metabolomics with matrix free laser/desorption ionization-MS to address microalgal physiology

**Tim U.H. Baumeister^1^, Marine Vallet^1^, Filip Kaftan^2^, Ales Svatos^2*^, Georg Pohnert^1,3*^**

^1^ Research Group Plankton Community Interaction, Max Planck Institute for Chemical Ecology, Jena, Germany

^2^ Research Group Mass Spectrometry/Proteomics, Max Planck Institute for Chemical Ecology, Jena, Germany

^3^ Institute for Inorganic and Analytical Chemistry, Bioorganic Analytics, Friedrich Schiller University Jena, Lessingstrasse 8, D-07743 Jena, Germany

*** Correspondence:**

Georg Pohnert: georg.pohnert@uni-jena.de

Ales Svatos: svatos@ice.mpg.de

**SI. Figure S1**. Metabolic comparison of LSC-MS profiles of two *C. granii* strains with unsupervised PCA. a) Strain SCCAP-K1834 and b) strain Helg2016. The ellipses represent the 95% confidence region.


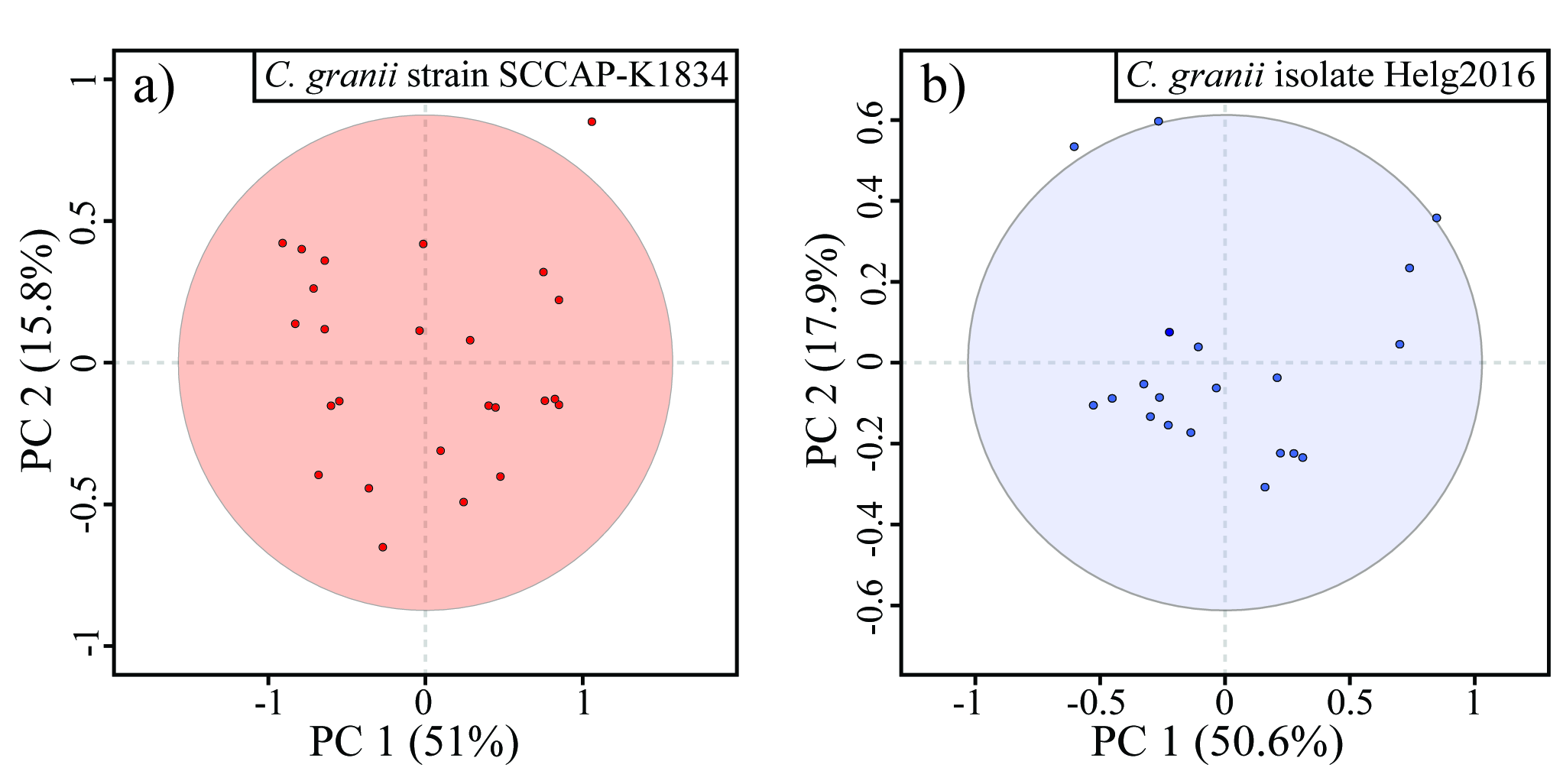


**SI. Table S1.** Shapiro-Wilk normality test to assess if observed peak intensities of prominent signals obtained from several analyzed microalgal cells follow a normal distribution. Selected signals were chlorophyll a (*m*/*z*892), fucoxanthin (*m*/*z*658), and β-carotene (*m*/*z*536). Diatom cells from the same culture at day 1 early growth of *Coscinodiscus granii* isolate Helg2016 (N = 20) and strain SCCAP-K1834 (N = 25).

| Shapiro-Wilk normality test | *C. granii* Helg2016  W (p-value) | *C. granii* SCCAP-K1834  W (p-value) |
| --- | --- | --- |
| Fucoxanthin *m*/*z*658 | 0.95371 (0.43) | 0.84326 (0.001) |
| β-carotene *m*/*z*536 | 0.94652 (0.32) | 0.82110 (<0.001) |
| Chlorophyll a *m*/*z*892 | 0.95299 (0.41) | 0.73622 (<0.001) |

**SI. Figure S2**. Microscopy picture and growth monitoring of diatoms *C. granii* (a) strain SCCAP-K1834 (b) isolate Helg2016.


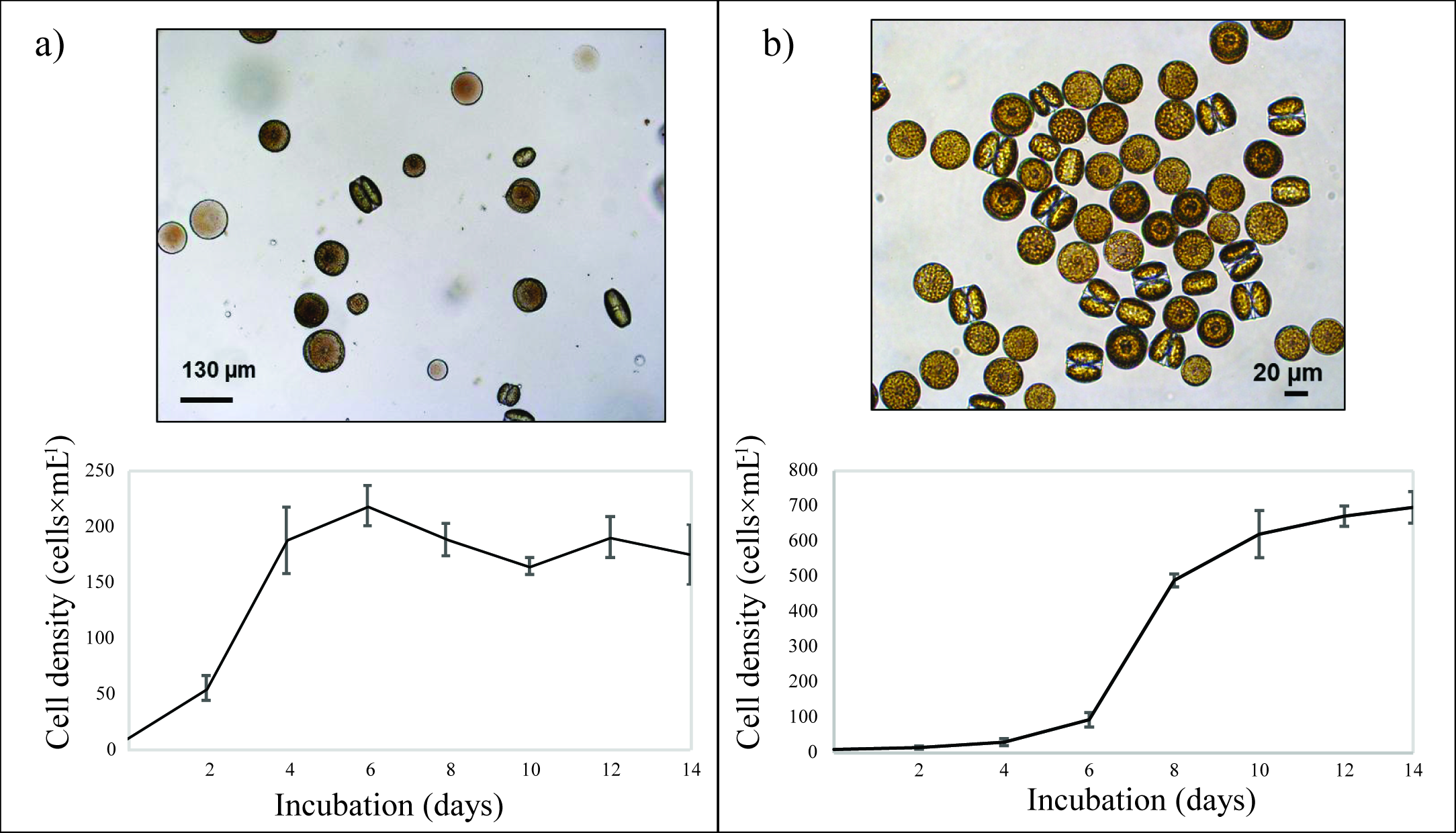


**SI. Figure S3**. Ions trace of DMSP in the LSC-MS spectrum of diatom *Coscinodiscus granii* strain SCCAP-K1834.


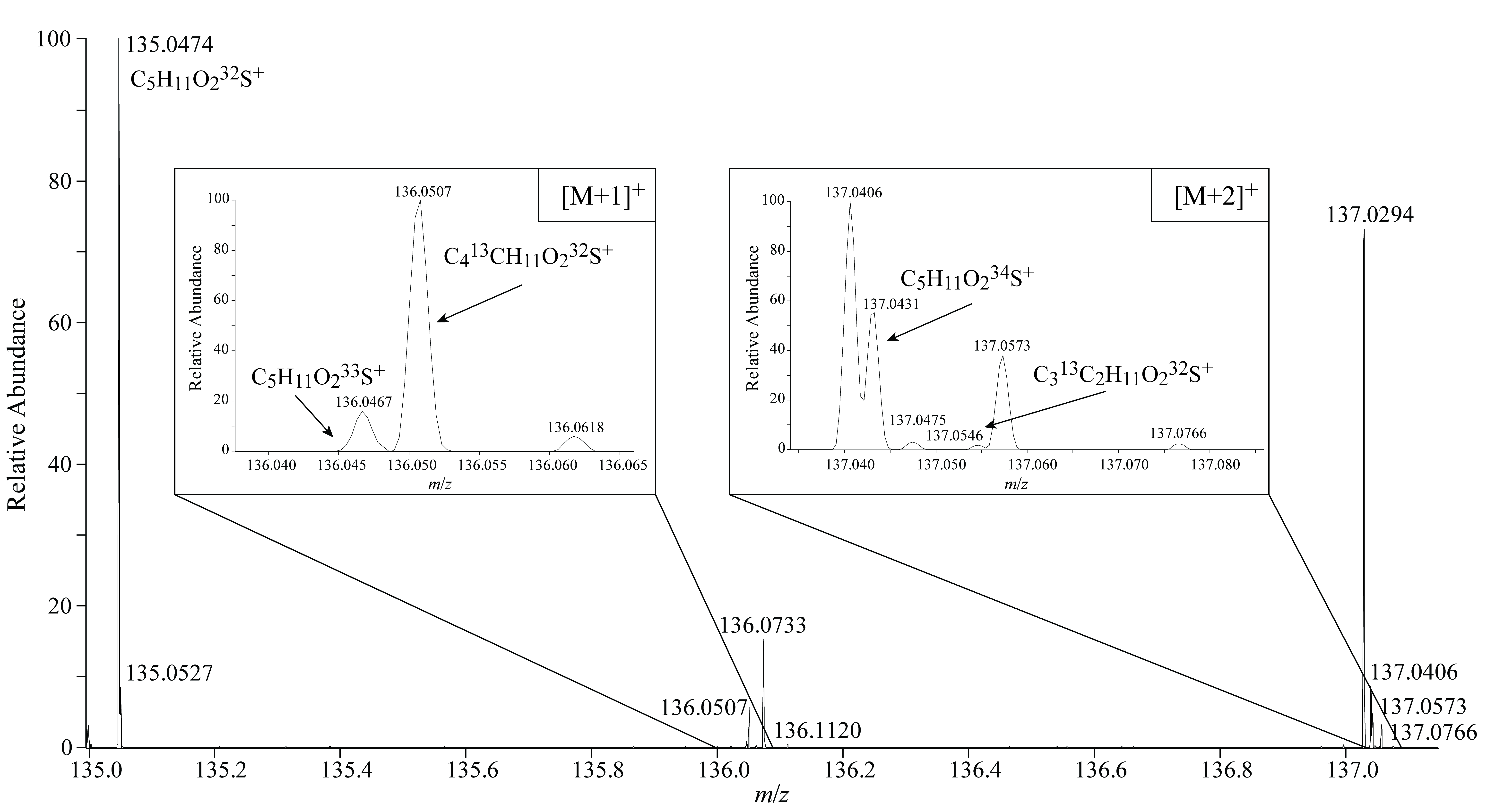


**SI. Figure S4.** PLS-DA classification assessed for two components. The stars indicates that components 1 and 2 are both accurate and reliable classifier. Q2 is an estimate of the predictive ability of the model, and is calculated via cross-validation (CV). For further details: <https://www.ncbi.nlm.nih.gov/pmc/articles/PMC3337399/>

**
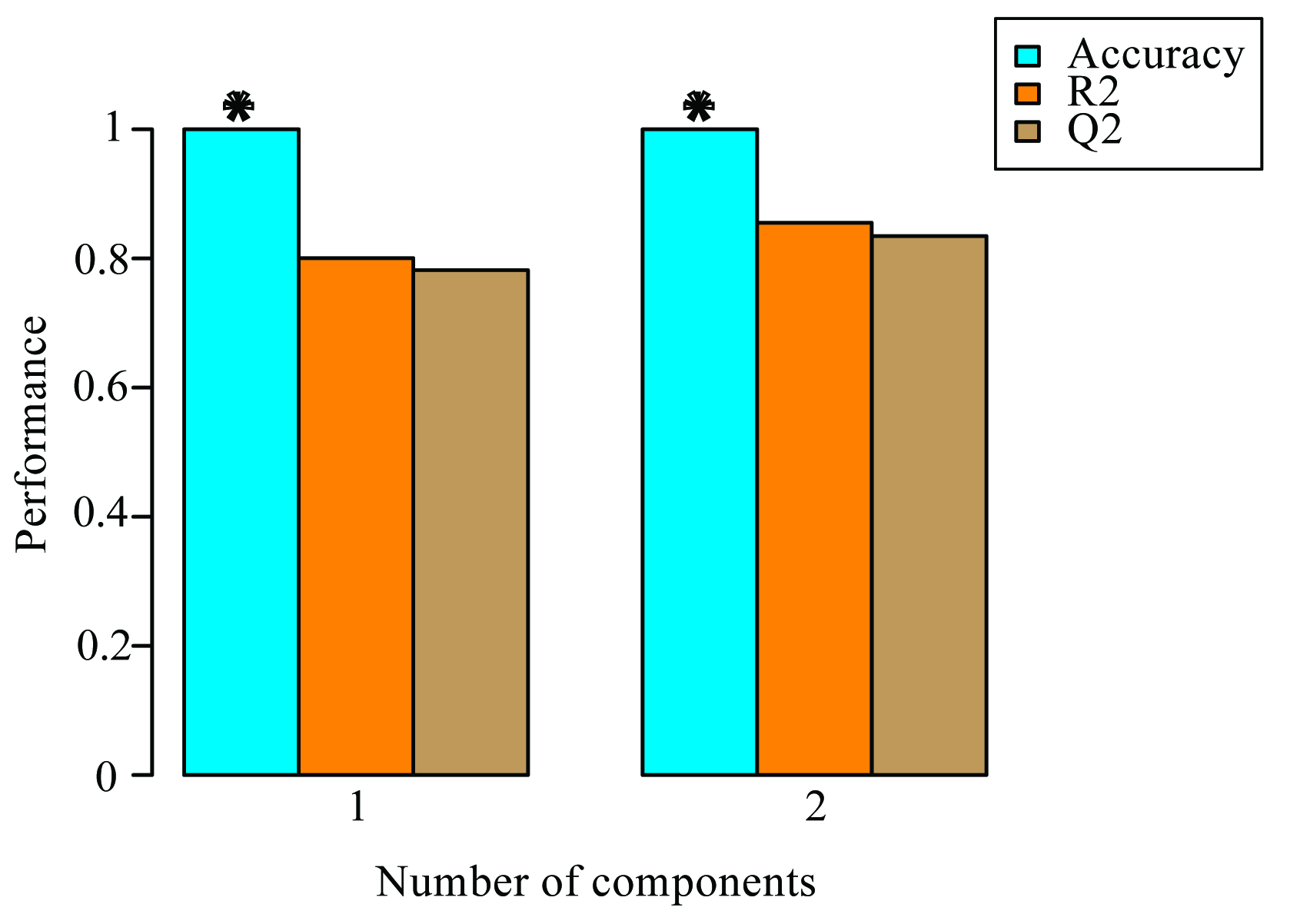
**

**SI. Figure S5.** PLSDA loading plot discriminating LSC-MS profiles of *Haematococcus pluvialis* cells in early and late growth phase.


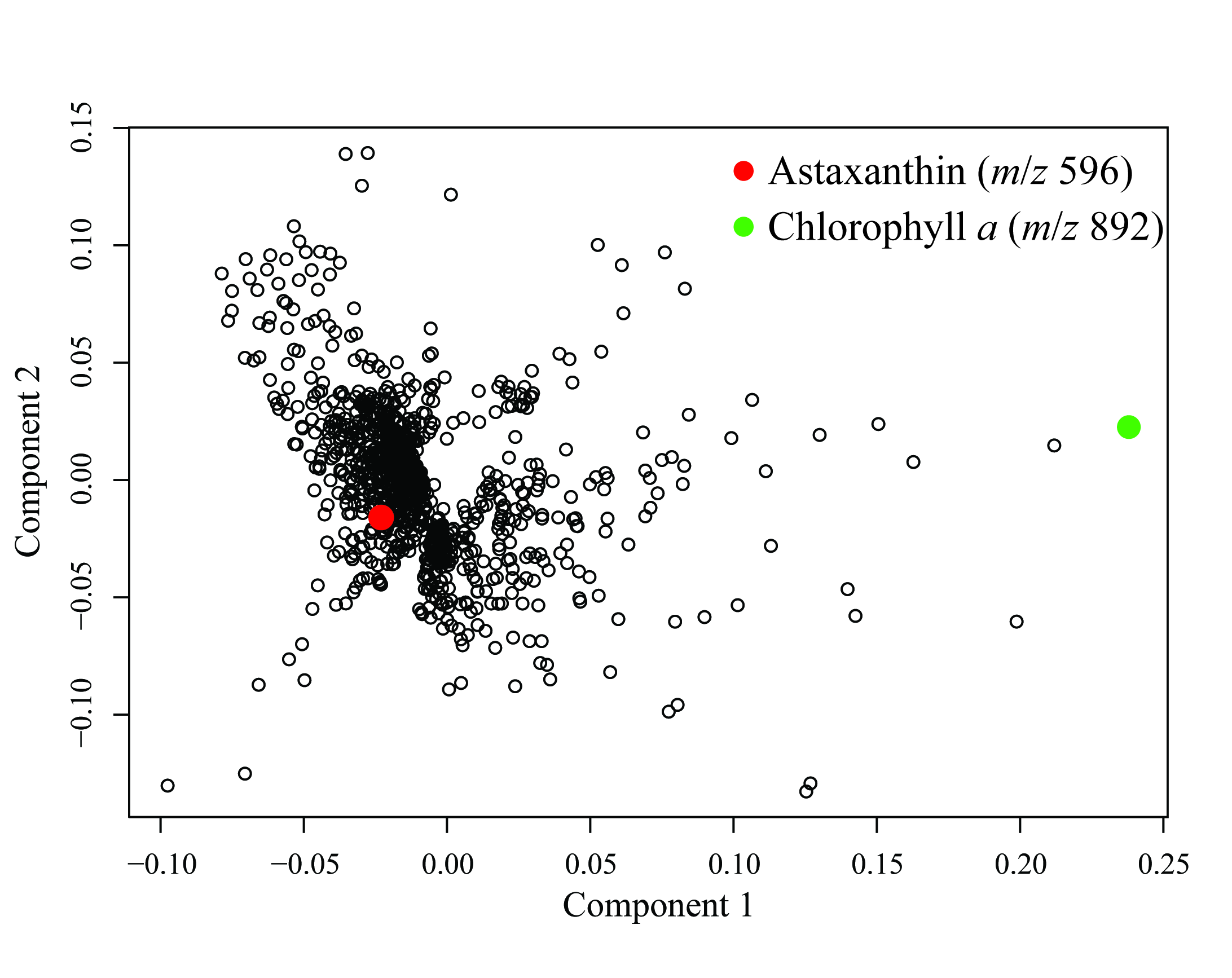


**SI. Figure S6.** Statistical differences found by paired t-test for chlorophyll a (*m*/*z* 892), β-carotene (*m*/*z* 536) and astaxanthin (*m*/*z* 596) ions trace detected in the full dataset of LSC-MS of *Haematococcus pluvialis* in early and late growth phases.


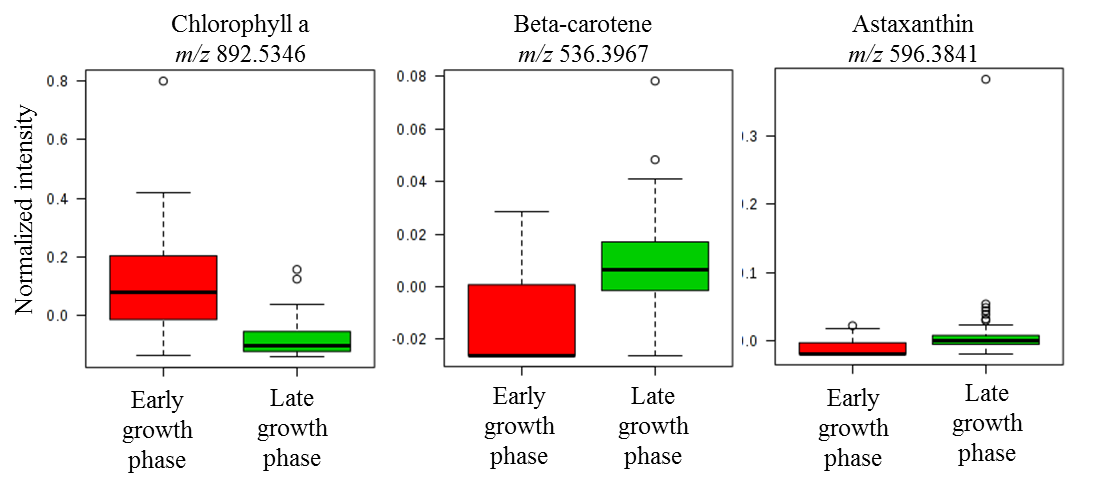

Supplement: Supplementary file 1 [file Data_Sheet_1.docx]
